# Data integration of National Dose Registry and survey data using multivariate imputation by chained equations

**DOI:** 10.1371/journal.pone.0261534

**Published:** 2022-06-15

**Authors:** Ryu Kyung Kim, Young Min Kim, Won Jin Lee, Jongho Im, Juhee Lee, Ye Jin Bang, Eun Shil Cha

**Affiliations:** 1 Department of Statistics, Kyungpook National University, Daegu, South Korea; 2 Department of Preventive Medicine, College of Medicine, Korea University, Seoul, South Korea; 3 Department of Statistics and Data Science, Yonsei University, Seoul, South Korea; 4 Department of Applied Statistics, Yonsei University, Seoul, South Korea; 5 Korea Disease Control and Prevention Agency, Cheongju, Chuncheongbuk-do, South Korea; University of Washington, UNITED STATES

## Abstract

**Introduction:**

Data integration is the process of merging information from multiple datasets generated from different sources, which can obtain more information in comparison to to one data source. All diagnostic medical radiation workers were enrolled in National Dose Registry (NDR) from 1996 to 2011, linked with mortality and cancer registry data. (https://kdca.go.kr/) Survey was conducted during 2012-2013 using self-reported questionnaire on occupational radiation practices among diagnostic medical radiation workers.

**Methods:**

Data integration of NDR and Survey was performed using the multivariate imputation by chained equations (MICE) algorithm.

**Results:**

The results were compared by sex and type of job because characteristics of target variables for imputation depend on these variables. There was a difference between the observed and pooled mean for the frequency of interventional therapy for nurses due to different type of medical facility distribution between observed and completed data. Concerning the marital status of males and females, and status of pregnancy for females, there was a difference between observed and pooled mean because the distribution of the year of birth was different between the observed and completed data. For lifetime status of smoking, the percentage of smoking experience was higher in the completed data than in the observed data, which could be due to reasons, such as underreporting among females and the distribution difference in the frequency of drinking between the observed and completed data for males.

**Conclusion:**

Data integration can allow us to obtain survey information of NDR units without additional surveys, saving us time and costs for the survey.

## Introduction

The use of medical radiation is on the rise due to the public’s increased interest in improving their quality of life and health care. Diagnostic medical radiation workers work in the field of diagnostic radiology, such as the management, operation, and control of a radiation generating device for diagnosis, and they are exposed to radiation in such jobs. The number of diagnostic medical radiation workers has increased consistently in South Korea and the importance of safety management for diagnostic medical radiation workers is also increasing [1]. Accordingly, studies on the effects of radiation exposure are ongoing to monitor lifetime occupational exposure of diagnostic medical workers to radiation [2–6].

Radiation exposure doses are measured quarterly to prevent potential health risks to diagnostic medical radiation workers. Also, the information that can be used as surrogate measurements of occupational radiation exposure are demographic factors, work history, work practices, lifestyle factors, etc [3]. There are eight occupations that are associated with diagnostic medical radiation workers (category: radiological technologist, radiologist, dentist, dental hygienist, nurse, doctor, assistant, and the others), with different level of exposure doses for each occupation.

For example, imagine a scenario where the data on demographic information in data set A and work history information in data set B are separate, not linked and does not exist in a single data set. Also imagine another scenario where units in data A (unit A) and the units in data B (unit B) are not the same and the unit B is part of the unit A. If the two datasets are not linked, it would be impossible to conduct analysis using demographic information and work history information together. In addition, linking two data while all the units do not match makes the joint information available only for the common units. This makes the information in some unit A not linked unavailable and results in loss of information. Data integration can provide a variety of available information, improve the quality of data, and expand the domain of research.

The purpose of this paper is to generate a unified single dataset by applying data integration to multiple sources associated with radiation factors for diagnostic medical radiation workers.

## Materials and methods

### National Dose Registry (NDR)

All 94,394 diagnostic medical radiation workers were enrolled in the radiation dose registry from 1996 to 2011. Within the registry, the information in the workers’ records included demographic factors (e.g., name, sex, date of birth, type of job, type of medical facility, area of medical facility, location of medical facility), quarterly dose information for individuals, and the beginning and the end of the period of dose measurement. The National Cancer Center administers the Korea Central Cancer Registry data, which includes status of cancer, cancer code, site, stage, and date of diagnosis. The cancer registry data were followed up through 2017. Statistics Korea provides mortality data, which were followed up through 2018. The data included status of death, date of death, cause, and type of job. The radiation dose registry was linked with cancer registry and mortality data and the linked data was named the NDR [7].

94,379 units in NDR are considered for this data integration excluding people due to errors in date of birth.

### Associated survey (Survey)

The survey methods and baseline findings have been described previously [2]. Briefly, the associated survey (Survey) is a self-reported questionnaire survey via internet, on-site, visit, fax, and postal methods for diagnostic medical radiation workers between 2012 and 2013. There were three types of survey related to this study: four-page survey, two-page short form survey, and survey for dental workers. Four-page survey included demographic factors, exposure, and occupational history (e.g., starting year of work, duration of employment, frequency of wearing a dosimeter, frequency of using personal protective equipment, frequency of diagnostic procedures per week, radiation work history), lifestyle factors (e.g., smoking, drinking, marital status), reproductive and gynecological history (e.g., age at menstruation, status of menopause, age of menopause, status of pregnancy, the number of births). To increase the response rate, the four-page survey was modified to a two-page format featuring only questions about demographic factors and occupational radiation exposure. That is, questions about lifestyle factors and reproductive and gynecological history were excluded. Survey for dental workers excluded non-dental radiation work history, e.g., frequency of CT, C-Arm, fluoroscopy, and interventional therapy per week. This survey was reviewed and approved by the Institutional Review Board of Korea University (KU-IRB-12–12-A-1). Informed written consent was voluntarily obtained from all individual study participants prior to enrollment.

Survey included 15,501 people, but one person was excluded because the date of birth was wrongly recorded. Of the survey respondents, 12,906 people were linked with cancer registry data and mortality data in NDR. A total of 2,594 people were not linked with the data because of incorrect information (e.g., date of birth, name, etc.) or because they were not yet included in NDR. Of the 2,594 people that were not linked, there were two units whose date of birth and names were similar to those in NDR. Comparing this with other information, the two were determined to be the same people in NDR and Survey. Hence 12,908 units in Survey were considered.

Detailed information regarding the survey and questionnaire used in this study can be obtained previous published article [2].

### Data integration and imputation

Data integration including statistical matching, and record linkage is the process of merging information from several heterogeneous datasets [8–12]. Data integration and analysis using linked data have been steadily studied in epidemiology [13–15]. Data integration has the advantage of enabling users or researchers to implement statistical analysis for variables that cannot be jointly observed within each dataset. For example, when a contingency table of a variable *X* in data A and a variable *Y* in data B are required, it is not possible to produce the contingency table if the two data are not integrated and the two variables are not in the same dataset. Data integration can provide a variety of available information and expand the domain of research. Also, it improves the data quality better than if only one data source (limited information) is considered.

Data integration can be implemented in two ways. One is macro-level data integration, which is the process of merging datasets from different sources on the aggregate level to allow a coherent analysis of the data. Macro-level data integration is “balancing” of inconsistent statistical data within the limits of identities which must be fulfilled and a plausible outcome [16]. The other is micro-level data integration which is the process of generating a unified single dataset by “combining” datasets from different sources on the unit level.

Data integration relates to the missing data problems. It considers non-common variables to missing. Thus, for data integration, it is necessary to understand the missing data mechanism. There are three categories in missing mechanisms: MCAR, MAR and MNAR. Let (**x**, *Y*, *δ*) be the data unit where **x** is a covariates vector, *Y* is a study variable and *δ* is a missing indicator whether *Y* is observed. If the missing mechanism does not depend on the covariates **x** and study variable *Y*, i.e.,

P(δ=1|x,Y)=P(δ=1),

then the data are called missing completely at random (MCAR). When the data are missing at random (MAR), the probability that occurs the missing does not depend on the study variable *Y* but depends on covariates **x**:

P(δ=1|x,Y)=P(δ=1|x).

Finally, if the data are missing not at random (MNAR), the probability of missing depends on *Y*. It should be used appropriate method according to these mechanisms [19, 20].

On the other hands micro-level data integration uses editing and imputing techniques to make the integrated datasets more consistent for the data integration from combined sources [9]. One simple way of implementing data integration is to use imputation methods. Imputation methods can be categorized into single imputation and repeated imputation depending on the size of imputed values in each missing value. It is called single imputation when one imputed value is used for filling in each missing value, whereas repeated imputation produces multiple imputed values for each missing value [17, 18]. In practice, repeated imputation is more recommended than single imputation, because it is not easy to preserve the variance-covariance structure with single imputation [18].

There are two generally mentioned repeated imputations: multiple imputation and fractional imputation. Multiple imputation, proposed by Rubin [19, 20], conducts the imputation for some values multiple times to create *m* completed data, *Y*_*com*_ = (*Y*_*obs*_, *Y*_*imp*_), and *m* denotes the number of imputations. Fractional imputation, initially proposed by Kalton and Kish [21], generates a single completed data with fractional weights after imputation. The size of the completed data is always larger than the size of the original incomplete data. Compared to fractional imputation, multiple imputation is more popularly used in practice because it is easier to obtain the variance of the estimator.

We used multiple imputation method in this study. The aim of multiple imputation is to provide statistically valid inference [22]. We can obtain the *m* estimators ${\hat{Q}}_{1},\ldots ,{\hat{Q}}_{m}$ which denote completed data estimates for the quantity of research interest, e.g., a regression coefficient, by analyzing *m* times using the method that we would have used if the data had been completed. ${\bar{U}}_{i}$ implies the associated variance for an estimated parameter *Q*. The *m* estimators are pooled into one estimator $\bar{Q}=\frac{1}{m}\sum_{i=1}^{m}{\hat{Q}}_{i}$ and their variances are estimated using Rubin’s rules [20].

The pooling step using Rubin’s rules is as follows. Let *ν*_*com*_ be the degrees of freedom for approximate or exact *t*–test inferences about *Q* when there are no missing values [23, 24]. Denote $\bar{U}$ as within imputation variance and *B* as between imputation variance. Then, the total variance of $\bar{Q}$ is

$$T=\bar{U}+B+\frac{B}{m},$$

where

$$\begin{array}{ccc} \bar{U} & = & \frac{1}{m}\sum_{i=1}^{m}\bar{U_{i}}\;\text{and}\\ B & = & \frac{1}{m-1}\sum_{i=1}^{m}\left(\hat{Q_{i}}-\bar{Q}\right){\left(\hat{Q_{i}}-\bar{Q}\right)}^{T}. \end{array}$$

In total variance *T*, $\bar{U}$ is the variance due to taking samples rather than the entire population, *B* is the extra variance due to missing in sample, and *B*/*m* is the extra variance due to the fact that $\bar{Q}$ is estimated for finite *m* [25]. To evaluate the appropriateness of the data integration with NDR and Survey, we considered the (1-*α*)100% confidence interval [23, 26] which was computed as

$$(\bar{Q}-t_{\nu_{adj}}\left(\alpha /2\right)\sqrt{T},\;\bar{Q}+t_{\nu_{adj}}\left(\alpha /2\right)\sqrt{T}),$$

where

$$\begin{array}{ccc} \nu_{adj} & = & \nu_{old}\times \nu_{obs}/\left(\nu_{old}+\nu_{obs}\right),\\ \lambda & = & (B+B/m)/T,\\ \nu_{old} & = & \left(m-1\right)/\lambda^{2},\;\text{and}\\ \nu_{obs} & = & \nu_{com}\left(1-\lambda \right)\left(\nu_{com}+1\right)/\left(\nu_{com}+3\right). \end{array}$$

Note that λ is the proportion of missing information for the unknown quantity of research interest, *ν*_*old*_ [20, 26] is the generally used degree of freedom for student’s *t*-distribution, and *ν*_*obs*_ is the estimated observed data degrees of freedom. *ν*_*old*_ can produce values greater than the sample size in the entire data, and to solve this inappropriate situation, the refined degree of freedom *ν*_*adj*_ was proposed by Barnard and Rubin [23]. The expression for *ν*_*adj*_ shows that *ν*_*adj*_ is always less than or equal to both *ν*_*old*_ and *ν*_*obs*_.

### Multivariate Imputation by Chained Equations (MICE)

In this study, we assume the missing at random. MICE is one of the multiple imputation techniques, which can be applied to data under missing at random (MAR) and missing not at random (MNAR) assumption [27, 28]. MICE is based on variable-by-variable basis and imputes missing data multiple times in a dataset through an iterative procedure. The MICE starts by first filling in the missing values randomly or with the desired values. The missing values are then imputed in the order of columns in the data or sequentially in the preset order. Van Buuren and Groothuis-Oudshoorn provided R package for MICE [28] which we used for the data integration in this paper.

## Results

### Data integration of NDR and survey

A specific purpose of this study was to obtain Survey information for NDR units, which were not included in Survey. There were many variables collected only in Survey, of which approximately 30 were set as target variables that required to be imputed: Demographic factors (height, weight); Exposure and employment history (starting year of work, frequency of wearing a dosimeter, wearing a lead apron, location of the dosimeter, frequency of work separated from patients completely, frequency of work standing behind a shield wall, frequency of holding patients, wearing a thyroid shield, lead goggle, lead gloves, and frequency of CT, C-Arm, fluoroscopy, and interventional therapy per week); Lifestyle factors (lifetime status of smoking, age at starting smoking, current status of smoking, current amount of smoking, age at quitting smoking, past amount of smoking, current status of drinking, frequency of drinking, amount of drinking, status of exercise, frequency of exercise, hours of sleep, marital status, and duration of shift work); Reproductive and gynecological history (age at menstruation, status of menopause, age at menopause, status of pregnancy, the number of births). There were missing parts in type of job, so type of job was also included in the target variable.

**Fig 1:**
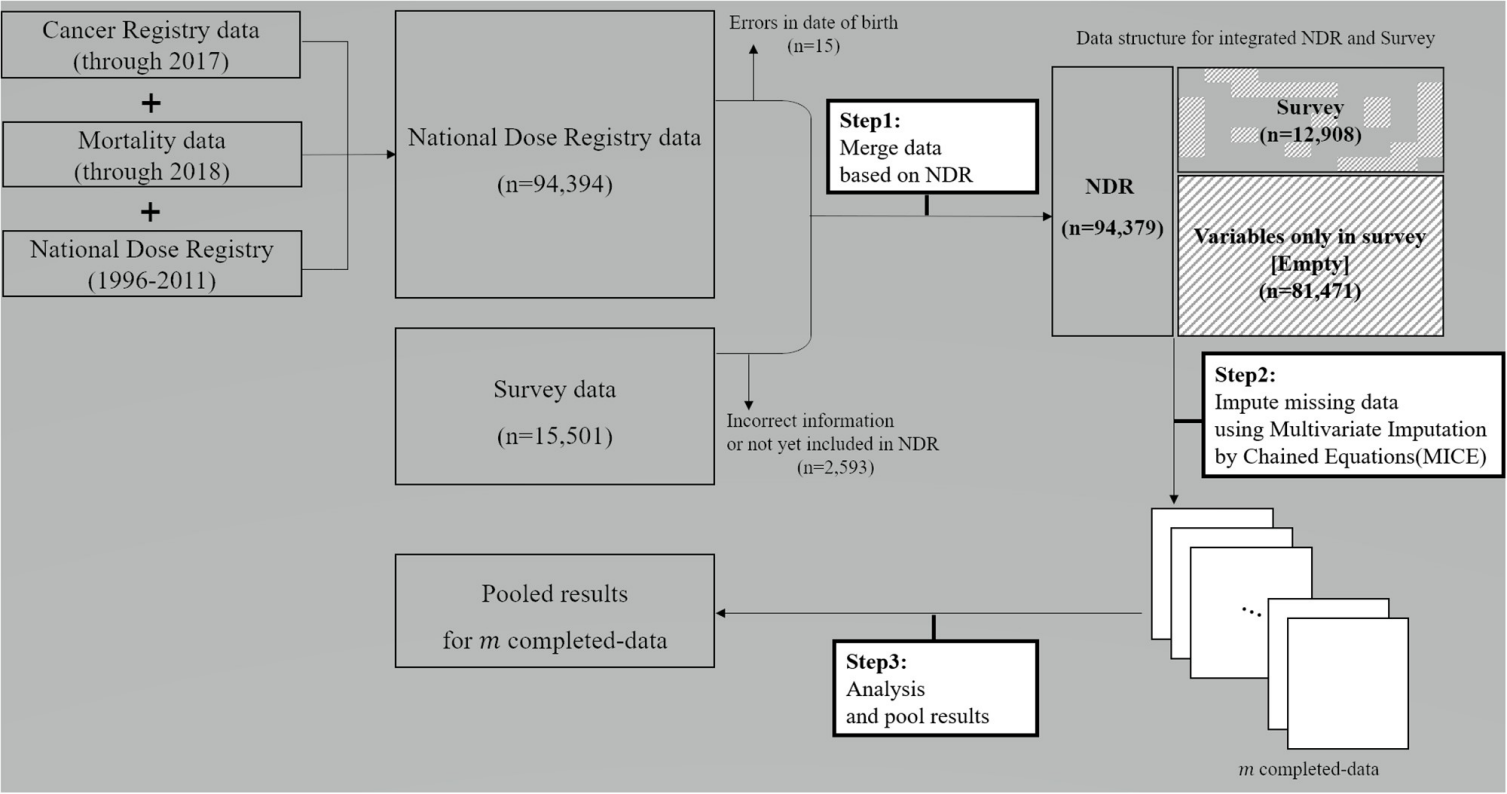
Overview of the data integration process.

First step, for data integration, we merged NDR and Survey based on NDR individuals, which means all records from NDR (94,379), and the matched records from the Survey (12,908). For units that existed only in NDR (81,471), the variables collected only in Survey were unobserved and emptied in integrated data in Fig 1. Empty values were considered as unit non-response and missing values were considered as item non-response in Survey. The radiation dose is the main factor in radiation epidemiology, and the NDR data has the covariate. Thus, the data integration only focused on the NDR dataset side. Thus, 2,594 units in the Survey dataset were not linked. As the next step, we imputed target variables using the MICE algorithm.

It should be noted that since Survey is not a sampling data, there may be variables that differ from the distribution in NDR. The variable with the biggest difference was the year of birth, and the range in NDR was from 1906 to 1993 while the range in Survey was from 1930 to 1990.

There were six common variables (e.g., sex, year of birth, type of job, type of medical facility, location of medical facility, area of medical facility) that exist in both NDR and Survey. These variables were included in the predictors because they were correlated with the target variables.

In survey, respondents to a particular question are often determined by the previous one. Depending on this feature, the target variables were divided into three variables: (i) a flag variable, which refers to the variable that determines whether the unit is the subject of subsequent question (e.g., status of drinking), (ii) a resultant variable, which refers to the subsequent question affected by the response according to the flag variable (e.g., frequency of drinking for drinker), (iii) a general variable, which refers to the variable that is neither flag nor resultant variable.

We assumed that the missing was not based on the variable itself, but can be estimated through other variables. Therefore, the analysis was conducted under MAR assumption [29].

We set the order in which variables were imputed as the order of low missing rates, and then imputed the flag variable and the resultant variable in order. The number of iterations for imputation was set to 15 in consideration of computing time and efficiency [30, 31]. The results were compared by sex and type of job because characteristics of the target variables varied with sex and type of job. Thus, the type of job was imputed only once to make the data without missing type of job, and the subsequent imputation was conducted 20 times sequentially for that data. The suspected outliers were set as missing and returned to original value from the completed data.

We used the term “non-applicable” for the cases that the unit was not the respondent and therefore it was “unable” to answer. This term is different from “missing” because missing means the case that the unit is the respondent but “does not” answer. There were two cases of non-applicable: according to the type of survey (case 1) and the flag variable (case 2). For example, (#) questions were excluded from type # survey, so the respondent to these questions is set “non-applicable”. In addition, the frequency of drinking was a resultant variable affected by the response of the status of drinking which indicates a flag variable. Since the frequency of drinking was defined only for drinkers and was not imputed for non-drinkers, the frequency of drinking was non-applicable for non-drinkers.

The predictors for target variables were based on three types of criteria: (1) the absolute value of Cramer’s *V* above 0.3, (2) the absolute value of correlation coefficient above 0.3, and (3) p-value in one-way ANOVA under 0.1. We did not select variables that implied (case 1) non-applicable as the predictor because the type of survey was the external factor rather than the feature of the respondents.

We were particularly interested in the starting year of work. In the survey, there was a short-answer question for the starting year of work, and in NDR, the beginning of the period of dose measurement was a similar concept to that question. In computing radiation exposed dose, the beginning of the period of dose measurement as the starting year of work is important. However, It is difficult to identify the exact starting year of work for people who worked before 1996 because NDR registration began in 1996. Therefore, we assumed the short-answer response in the survey as true values and estimated the starting year of work based on that survey variable. Instead of imputing the starting year of work itself, we transformed year into age. In other words, we applied reparameterization to better estimate the starting year. Because NDR and Survey illustrate similar distribution for age at starting work but different distribution for year of birth (the units in NDR tend to be older than survey), it was possible that the imputed starting year of work in NDR was greater than the actual value. We computed the starting year of work from the age at starting work after imputing the age at starting work.

There were age variables in the data and the post-process was applied to them to meet the following logical criteria: (i) the imputed age variables cannot be more than age at survey, (ii) the imputed age at starting smoking is set to be lower than the age at quitting smoking, and (iii) the imputed age at menstruation is set to be lower than the age at menopause.

Details of the application for data integration have been reported in a previous material [32].

### Comparison of observed and completed data

The results were compared by sex and type of job because characteristics of the target variables varied with the sex and type of job. The contingency table of sex and type of job is depicted in Table 1. “N: obs” is the number of observed units and “N: obs+imp” is the number of completed (observed + imputed) units.

**Table 1:** The contingency table of sex and type of job.

	**Radiological technologist**		**Radiologist**		**Dentist**		**Dental hygienist**	
	N: obs	N: obs+imp	N: obs	N: obs+imp	N: obs	N: obs+imp	N: obs	N: obs+imp
Male	17250	17606	1193	1226	12269	12409	75	192
Female	9047	9186	544	567	3413	3557	13439	13502
	**Nurse**		**Doctor**		**Assistant**		**Other**	
	N: obs	N: obs+imp	N: obs	N: obs+imp	N: obs	N: obs+imp	N: obs	N: obs+imp
Male	424	501	15912	16101	5478	5674	145	153
Female	7144	7220	2643	2800	361	364	3178	3260

If 70 units were observed and 30 were missing in a typical imputation, the number of units completed is 100. However, for the Survey data, there was “non-applicable” category, and the “non-applicable” category was excluded from the estimation, so the number of completed units depended on the frequency of the flag variable in imputed data. Therefore, we set the mean for frequency of the resultant variables excluding “non-applicable” counts from the 20 completed data as the number of completed units. “obs mean” is the mean for numeric variables and the relative frequency for factor variables, “pooled mean” is the pooled mean for the *m* completed data by Rubin’s rule, and “95% CI” is the 95% confidence interval for the pooled mean.

All analysis results are provided as S1 File (see Table, Supplemental Digital Content 1, which provides all analysis results for all target variables) and only the results for the key variables are described in Table 2.

**Table 2:** Pooled mean and confidence interval (CI).

	N: obs	N: obs+imp	obs mean	pooled mean	95% CI
Age at starting work (other job)	121	8925	25.04	25.73	(25.15, 26.31)
Starting year of work (other job)	121	8925	2003.11	1997.22	(1996.61, 1997.83)
Wearing a lead apron (radiological technologist)					
0%	2569	7223	0.25	0.27	(0.26, 0.28)
< 25%	3254	8662	0.32	0.32	(0.32, 0.33)
25–74%	1061	2672	0.10	0.10	(0.09, 0.10)
≥ 75%	3293	8229	0.32	0.31	(0.30, 0.31)
Wearing a lead apron (nurse)					
0%	40	2236	0.17	0.29	(0.27, 0.30)
< 25%	19	673	0.08	0.09	(0.08, 0.10)
25–74%	11	409	0.05	0.05	(0.05, 0.06)
≥ 75%	169	4408	0.71	0.57	(0.55, 0.59)
Frequency of interventional therapy (nurse)					
Never	115	4532	0.43	0.59	(0.57, 0.60)
< 1	4	91	0.02	0.01	(0.01, 0.02)
1–4	8	205	0.03	0.03	(0.02, 0.03)
5–10	24	587	0.09	0.08	(0.07, 0.08)
11–14	5	173	0.02	0.02	(0.02, 0.03)
≥ 15	110	2138	0.41	0.28	(0.26, 0.29)
Marital status (male)					
Unmarried	871	6646	0.25	0.14	(0.13, 0.14)
Married	2575	40976	0.74	0.85	(0.84, 0.85)
Other	52	836	0.01	0.02	(0.02, 0.02)
Status of pregnancy (female)					
No	930	18874	0.59	0.49	(0.49, 0.50)
Yes	634	19277	0.41	0.51	(0.50, 0.51)
Status of drinking (male)					
No	592	11815	0.17	0.24	(0.21, 0.28)
Yes	2957	36643	0.83	0.76	(0.72, 0.79)
Frequency of drinking (male)					
Less than once a month	202	4016	0.07	0.11	(0.10, 0.11)
Once a month	271	4187	0.09	0.11	(0.11, 0.12)
2–4 a month	1449	17151	0.49	0.47	(0.46, 0.48)
2–3 a week	892	9838	0.30	0.27	(0.26, 0.28)
More than 4 a week	132	1450	0.04	0.04	(0.04, 0.04)
Lifetime status of smoking (female)					
No	1850	27213	0.99	0.71	(0.64, 0.77)
Yes	27	11265	0.01	0.29	(0.23, 0.36)
Lifetime status of smoking (male)					
No	1087	20035	0.31	0.41	(0.37, 0.46)
Yes	2450	28423	0.69	0.59	(0.54, 0.63)

Note: obs: observed data, imp: imputed data

**Fig 2:**
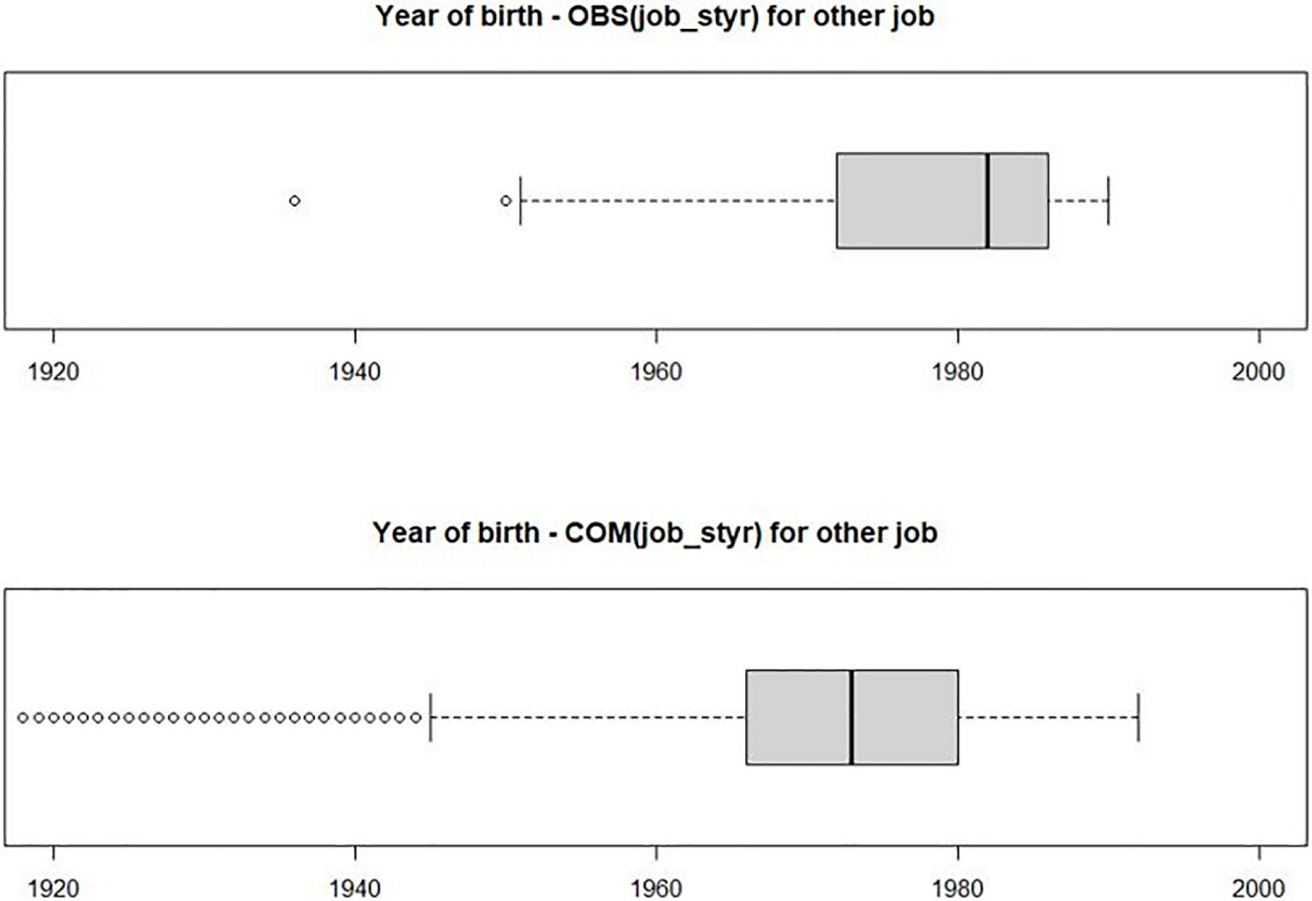
Box plot of year of birth by observed and completed group for starting year of work (other job). Note: OBS: observed data, COM: complete data which combines observed and imputed datasets.

In age at starting work and starting year of work for other job, “obs mean” and “pooled mean” for age were very similar, but for the year, there was a difference of approximately 6 years. In Fig 2, we can see that birth year of observed group tends to be smaller than completed group. These results are reasonable because the earlier the birth year, the lower the starting year of the work. There were also cases of distribution differences in assistant, which can also be attributed to differences in distribution in the year of birth.

Pertaining to wearing a lead apron for radiological technologist, “N: obs” were sufficient and “obs mean” and “pooled mean” were very similar, so imputation is reliable.

With regards to wearing a lead apron for nurses, “0%” was more imputed than observed and “≥ 75” was less imputed than observed. The proportion of nurses working in dental clinics was higher in the completed group than in the observed group. Most of dentists and dental hygienists did not wear a lead apron. Since nurses worked mostly in general hospitals and dental clinics, it seems reasonable that 0% category were frequently selected as imputed values.

In the frequency of interventional therapy for nurses, “Never” was more imputed than observed and “≥ 15” was less imputed than observed. The majority of dentists and dental hygienists in dental clinics answered “Never” in frequency of interventional therapy. Thus, since nurses usually worked in general hospitals and dental clinics, it seems reasonable that “Never” values were more imputed than observed.

**Fig 3:**
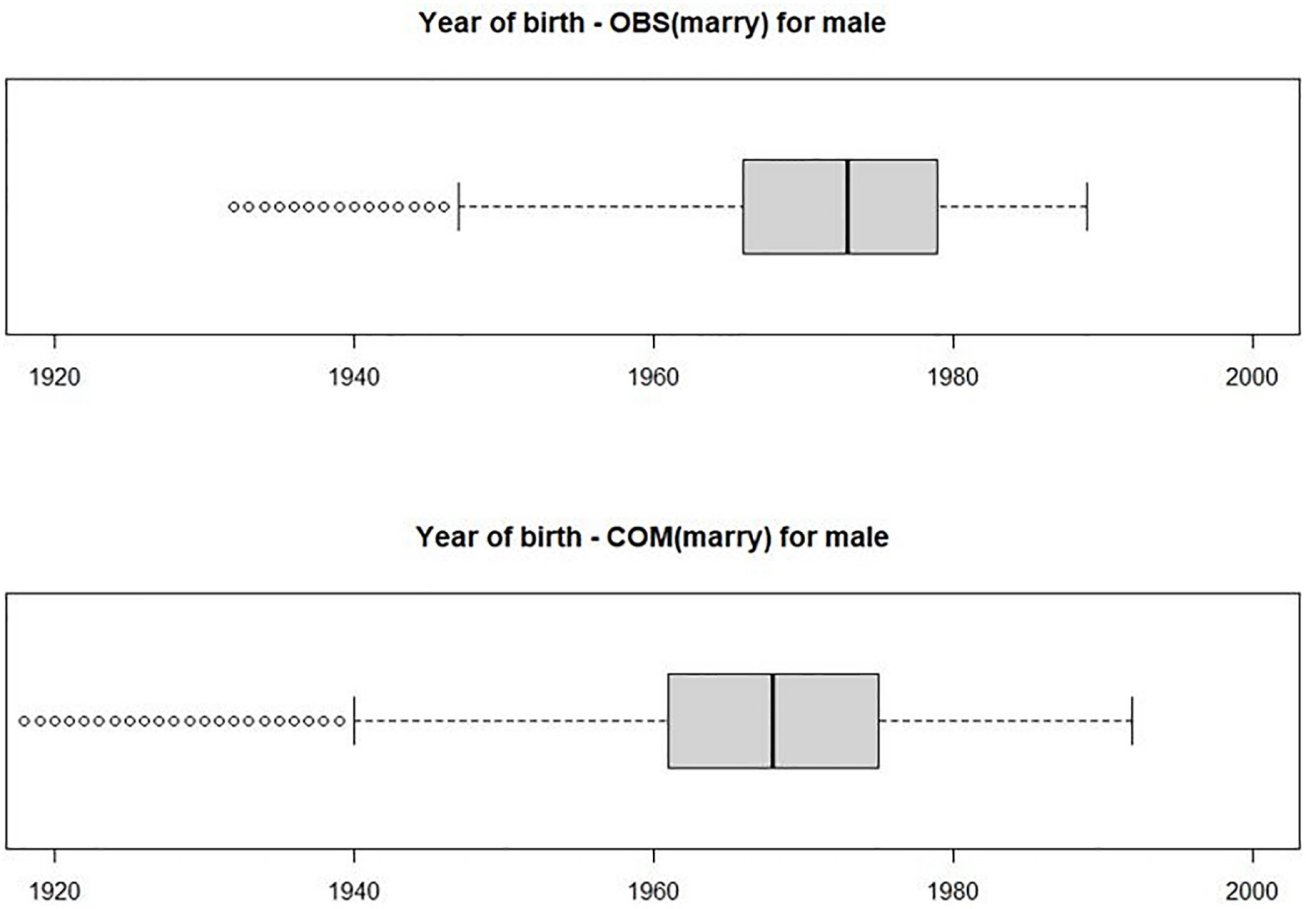
Box plot of year of birth by observed and completed group for marital status (male). Note: OBS: observed data, COM: complete data which combines observed and imputed datasets.

In marital status for males, the married category was imputed more than observed. Year of birth for completed data tends to be greater than for observed data in Fig 3. Older people were more likely to be married, so it suggests a reliable result. For females, the category of married was also more imputed than observed, and this was as a result of differences in distribution of birth years.

In the status of pregnancy for females, category yes was more imputed than observed. Year of birth for completed data tends to be greater than for observed data. Older people were more likely to get pregnant, which suggests a reliable result.

In status and frequency of drinking for males, the sum of counts for frequency of drinking was 36,642 and it was almost the same as the count for status of drinking “yes”. (Since the rounded values were used, there may have been a slight difference between values.) This was because frequency of drinking is non-applicable for status of drinking “No”. That is, frequency of drinking was defined only for people who drink, so frequency of drinking was not imputed for them. In “N: obs”, the count of status of drinking “Yes” differed from the sum of the counts of frequency of drinking. This was because there existed units where status of drinking was observed but frequency of drinking was missing. For these units, we imputed the frequency of drinking value with a value other than non-applicable.

In lifetime status of smoking for females, the frequency of “Yes” in the completed data was much higher than in observed data, which may be due to the tendency to underreport. In lifetime status of smoking for males, the frequency of “No” in the completed data was much higher than in observed data. There was a correlation between frequency of drinking and status of smoking, and the number of people with high frequency of drinking in males was found to be less in the completed group than in the group where status of smoking was observed. Therefore, it seems reasonable that more responses that have no smoking experience were imputed.

## Discussion

Comparing the distribution of observed data and completed-data with sufficient observed sample size, they generally had similar distribution. Most observations for demographic factors and exposure and employment history variables were sufficient and the distribution of the observed and completed data for these variables were similar.

For the starting year of work for other job and assistant, there was a difference in the distribution between observed and completed data due to a difference in the distribution of year of birth in the two data.

In some cases related to occupational history, there were differences in the distribution of observed and completed data, which was attributed to the difference in distribution between the two data in occupational-related variables such as type of medical facility.

In lifetime status of smoking, there was a difference in observed and pooled mean, which seems to be due to reasons such as underreporting among females and the distribution difference in the frequency of drinking between the observed and completed data for males.

In this study, we conducted data integration of NDR and Survey data using MICE algorithm. As a result, we generated a unified single dataset for NDR and Survey, preserved data size and reflected uncertainty. Registry data typically have a small number of variables. If registry data is integrated with data from other sources, more information is available and various analyses are possible. Data integration also allowed us to obtain survey information about NDR units without additional surveys, saving us time and costs for the survey. Since we obtained survey information about units observed only in NDR, enabling analysis using various information from 94,379 people, we can conduct statistical analysis for variables not jointly observed. The data quality is improved in comparison to data which have limited information from only NDR or survey, and the domain of research is greatly expanded.

The study was applied to NDR for diagnostic medical radiation workers. Moreover, it is meaningful to suggest a model that can address the dilemmas of national registry data (entire population) and survey data (subpopulation) commonly encountered in public health in the future although we cannot consider 2594 on the Survey dataset side. The most important variable in this study is radiation dose, but data on the Survey side do not have this information.

If you wish to see more information about variables and related results after micro-level data integration, please see the S1 File.

## Supporting information

S1 File (PDF)
