# Correction: Data integration of National Dose Registry and survey data using multivariate imputation by chained equations

**DOI:** 10.1371/journal.pone.0272879

**Published:** 2022-08-04

**Authors:** Ryu Kyung Kim, Young Min Kim, Won Jin Lee, Jongho Im, Juhee Lee, Ye Jin Bang, Eun Shil Cha

There are errors in the Funding statement. The correct Funding statement is as follows: This research was supported by the National Research Foundation of Korea (NRF) grant funded by the Korean government (MSIT) (No. 2020R1A2C1008891). This research was also supported by the Human Resources Program in Energy Technology of the Korea Institute of Energy Technology Evaluation and Planning (KETEP) granted financial resources from the Ministry of Trade, Industry & Energy, Republic of Korea (No. 20204010600060).
