# Supplementary material for: Data integration of National Dose Registry and survey data using multivariate imputation by chained equations
Source: PLoS One. 2022 Jun 15;17(6):e0261534. doi: 10.1371/journal.pone.0261534 (PMC9200363; doi:10.1371/journal.pone.0261534)
Supplement: S1 File — (PDF) [file pone.0261534.s001.pdf]

Supporting Information:
Tables that provide all analysis results for all target variables

Ryu Kyung Kim[a], Young Min Kim[a], Won Jin Lee[b], Jongho Im[c]
Juhee Lee[a]; Ye Jin Bang[b], Eun Shil Cha[c]
Kyungpook National University[a]; Korea University College of Medicine[b];
Yonsei University[c]; Korea Disease Control and Prevention Agency[d]

DESCRIPTION OF VARIABLES

Common variables

There are six common variables that exist in both NDR and Survey. If the scale type is factor, the levels of factor are expressed together. S1 illustrates common variables used in the analysis.

Name	Description	Scale type
Sex	Sex	Factor(2)
Byr	Year of birth	Numeric
Job9_new	Type of job	Factor(8)
Mtype_new	Types of medical institutions	Factor(8)
Sido	Area of medical institutions	Factor(16)
Sgg	Location of medical institutions	Factor(3)

S1 Table Description of common variables

Target variables

In survey, respondents to a particular question are often determined by the previous one. Depending on this feature, the target variable can be divided into three variables:

i. The flag variable refers to the variable that determine whether the unit is the subject of subsequent question.

ii. The resultant variable refers to the subsequent question affected by the response according to the flag variable.

iii. The general variable refers to the variable neither flag nor resultant variable.

In S2 Table, S3 Table and S4 Table, flag variables are labeled (i), resultant variables are labeled (ii), and general variables are not labeled. For example, age at starting smoking(sm_stage) and current status of smoking(sm_cu) are defined only for smoker(i.e. sm_lt is 'Yes'), so sm_lt is labeled (i) and sm_stage and sm_cu are labeled (ii). Also, current amount of smoking(sm_cua) is defined only for current smoker(i.e. sm_cu is 'Yes'), so sm_cu is also labeled (i) and sm_cua is labeled (ii). For non-smoker(i.e. sm_lt is 'No'), sm_cu is set 'non-applicable'. Age at quitting smoking(sm_quage) and past amount of smoking(sm_paa) are defined only for ex-smoker(i.e. sm_cu is 'No'), so sm_cu is labeled (i) and sm_quage and sm_paa are labeled (ii).

There are three types of survey related to the analysis:

a. Survey for dental worker

b. Two-page short form survey

c. Four-page survey

The variable labeled (#) is the question excluded from the type # survey. For example, (a) labeled variables(e.g. Ct, Carm, Fluor, and Inter) are the questions excluded from the survey for dental worker.

Name	Description	Scale type
Job_styr	Starting year of work	numeric
Badge	Frequency of wearing a dosimeter	factor(3)
Apron$^{(i)}$	Frequency of wearing a lead apron	factor(4)
Badir$^{(ii)}$	Location of the dosimeter when wearing an apron	factor(3)
Sep$^{(i)}$	Frequency of work separated from the patient completely	factor(4)
Scr$^{(ii)}$	Frequency of work standing behind a shield wall	factor(4)
Hold$^{(ii)}$	Frequency of holding patients	factor(4)
Prot1	Frequency of wearing a thyroid shield	factor(4)
Prot2	Frequency of wearing a lead goggle	factor(4)
Prot3	Frequency of wearing a lead gloves	factor(4)
Ct	Frequency of CT per week$^{(a)}$	factor(6)
Carm	Frequency of C-Arm per week$^{(a)}$	factor(6)
Fluor	Frequency of fluoroscopy per week$^{(a)}$	factor(6)
Inter	Frequency of interventional therapy per week$^{(a)}$	factor(6)

S2 Table Description of target variables - exposure and occupational history

Name	Description	Scale type
Sm_lt$^{(i)}$	Lifetime status of smoking$^{(b)}$	factor(2)
sm_stage$^{(ii)}$	Age at starting smoking$^{(b)}$	numeric
sm_cu$^{(ii),(i)}$	Current status$^{(b)}$	factor(2)
sm_cua$^{(ii)}$	Current amount of smoking$^{(b)}$	numeric
sm_quage$^{(ii)}$	Age at quitting smoking$^{(b)}$	numeric
sm_paa$^{(ii)}$	Past amount of smoking$^{(b)}$	numeric
dr_cu$^{(i)}$	Current status of drinking$^{(b)}$	factor(2)
dr_fq$^{(ii)}$	Frequency of drinking$^{(b)}$	factor(5)
dr_a$^{(ii)}$	Amount of drinking$^{(b)}$	factor(5)
exer$^{(i)}$	Status of exercise$^{(b)}$	factor(2)
exer_fq$^{(ii)}$	Frequency of exercise$^{(b)}$	factor(4)
sl_hr	Hours of sleep$^{(b)}$	factor(5)
Ht	Height	numeric
Wt	Weight	numeric
Marry	Marital status$^{(b)}$	factor(3)
Shft_dur	Duration of shift work$^{(b)}$	factor(6)

S3 Table Description of target variables - lifestyle and demographic information

Name	Description	Scale type
mens_stage	Age at menstruation$^{(b)}$	numeric
meno_p12$^{(i)}$	Status of menopause$^{(b)}$	factor(2)
meno_page$^{(ii)}$	Age at menopause$^{(b)}$	numeric
preg$^{(i)}$	Status of pregnancy$^{(b)}$	factor(2)
para_num$^{(ii)}$	The number of births$^{(b)}$	numeric

S4 Table Description of target variables - reproductive and gynecological history

TABLES OF RESULTS FOR TARGET VARIABLES

The following pages provide complete tables of results for the target variables(Table **??**-**??**).

	Male					Female				
	N: obs	N: obs+imp	OBS mean	pooled mean	95% CI	N: obs	N: obs+imp	OBS mean	pooled mean	95% CI
Lifetime status of smoking										
No	1087	20035	0.31	0.41	(0.37, 0.46)	1850	27213	0.99	0.71	(0.64, 0.77)
Yes	2450	28423	0.69	0.59	(0.54, 0.63)	27	11265	0.01	0.29	(0.23, 0.36)
Age at starting smoking										
	2437	28470	20.34	21.02	(20.76, 21.27)	25	10880	20.40	21.06	(19.91, 22.22)
Current status of smoking										
	912	13402	0.37	0.47	(0.43, 0.51)	15	6954	0.60	0.64	(0.39, 0.88)
	1533	15069	0.63	0.53	(0.49, 0.57)	10	3926	0.40	0.36	(0.12, 0.61)
Current amount of smoking										
	1531	15069	12.31	12.31	(11.53, 13.08)	10	3926	10.30	9.42	(5.39, 13.45)
Age at quitting smoking										
	901	13402	35.66	39.84	(38.90, 40.79)	15	6954	25.67	31.14	(28.10, 34.18)
Past amount of smoking										
	902	13402	13.46	14.37	(13.32, 15.43)	15	6954	5.60	8.33	(4.99, 11.66)
Current status of drinking										
No	592	11815	0.17	0.24	(0.21, 0.28)	650	14500	0.34	0.38	(0.34, 0.41)
Yes	2957	36643	0.83	0.76	(0.72, 0.79)	1243	23978	0.66	0.62	(0.59, 0.66)
Frequency of drinking										
Less than once a month	202	4016	0.07	0.11	(0.10, 0.11)	198	4266	0.16	0.18	(0.17, 0.19)
Once a month	271	4187	0.09	0.11	(0.11, 0.12)	210	3796	0.17	0.16	(0.15, 0.17)
2-4 a month	1449	17151	0.49	0.47	(0.46, 0.48)	630	11041	0.51	0.46	(0.45, 0.47)
2-3 a week	892	9838	0.30	0.27	(0.26, 0.28)	181	4244	0.15	0.18	(0.17, 0.19)
$\geq$ 4 a week	132	1450	0.04	0.04	(0.04, 0.04)	16	632	0.01	0.03	(0.02, 0.03)
Amount of drinking										
1-2	194	5389	0.07	0.15	(0.14, 0.15)	239	4958	0.20	0.21	(0.20, 0.22)
3-4	501	8612	0.17	0.24	(0.23, 0.24)	381	7662	0.32	0.32	(0.31, 0.33)
5-6	677	8794	0.23	0.24	(0.23, 0.25)	292	6156	0.24	0.26	(0.24, 0.27)
7-9	773	7292	0.27	0.20	(0.19, 0.21)	178	2964	0.15	0.12	(0.12, 0.13)
$\geq$ 10	746	6556	0.26	0.18	(0.17, 0.19)	119	2236	0.10	0.09	(0.09, 0.10)
Status of exercise										
No	1715	19852	0.48	0.41	(0.39, 0.43)	1336	25702	0.71	0.67	(0.63, 0.71)
Yes	1828	28606	0.52	0.59	(0.57, 0.61)	549	12776	0.29	0.33	(0.29, 0.37)
Frequency of exercise										
1-2	784	11901	0.43	0.42	(0.41, 0.42)	208	5648	0.38	0.44	(0.43, 0.46)
3-4	764	12319	0.42	0.43	(0.42, 0.44)	254	5671	0.47	0.44	(0.43, 0.46)
5-6	199	3396	0.11	0.12	(0.11, 0.13)	75	1234	0.14	0.10	(0.08, 0.11)
Daily	72	991	0.04	0.03	(0.03, 0.04)	9	223	0.02	0.02	(0.01, 0.02)
Height										
	8852	53862	173.05	172.23	(171.95, 172.51)	3737	40517	161.32	160.94	(160.43, 161.44)
Weight										
	8833	53862	71.65	71.60	(71.18, 72.01)	3628	40517	53.18	53.14	(52.65, 53.64)
Marital status										
Unmarried	871	6646	0.25	0.14	(0.13, 0.14)	980	16460	0.52	0.43	(0.42, 0.43)
Married	2575	40976	0.74	0.85	(0.84, 0.85)	865	21407	0.46	0.56	(0.55, 0.56)
Other	52	836	0.01	0.02	(0.02, 0.02)	23	611	0.01	0.02	(0.01, 0.02)
Hours of sleep										
<5	80	1111	0.02	0.02	(0.02, 0.03)	40	858	0.02	0.02	(0.02, 0.02)
5-6	1052	13203	0.30	0.27	(0.27, 0.28)	619	14641	0.33	0.38	(0.37, 0.39)
7	1661	23677	0.47	0.49	(0.48, 0.49)	774	14534	0.41	0.38	(0.37, 0.38)
8	686	9448	0.19	0.19	(0.19, 0.20)	387	7262	0.20	0.19	(0.18, 0.19)
more than 9	74	1020	0.02	0.02	(0.02, 0.02)	69	1182	0.04	0.03	(0.03, 0.03)
Age at menstruation										
						1545	38151	14.72	14.80	(14.73, 14.87)
Age at menopause										
						55	2543	46.89	44.54	(42.97, 46.11)
Status of pregnancy										
No						930	18874	0.59	0.49	(0.49, 0.50)
Yes						634	19277	0.41	0.51	(0.50, 0.51)
The number of births										
						621	19277	1.62	1.66	(1.56, 1.77)

S5 Table Result of life style factors by sex

	Radiological technologist					Radiologist					Dentist					Dental hygienist				
	N: obs	N: obs+imp	OBS mean	Pooled mean	95% CI	N: obs	N: obs+imp	OBS mean	Pooled mean	95% CI	N: obs	N: obs+imp	OBS mean	Pooled mean	95% CI	N: obs	N: obs+imp	OBS mean	Pooled mean	95% CI
Job_styr																				
	11152	26792	2000.11	2000.33	(2000.20, 2000.46)	134	1793	1992.37	1992.54	(1991.92, 1993.16)	434	15966	1998.66	1997.01	(1996.26, 1997.76)	274	13694	2003.66	2003.15	(2002.78, 2003.52)
Wearng a lead apron																				
0%	2569	7223	0.25	0.27	(0.26, 0.28)	21	306	0.18	0.17	(0.15, 0.19)	207	9274	0.55	0.58	(0.57, 0.59)	195	10538	0.74	0.77	(0.76, 0.78)
<25%	3254	8662	0.32	0.32	(0.32, 0.33)	23	345	0.19	0.19	(0.16, 0.22)	80	3220	0.21	0.20	(0.19, 0.21)	32	1459	0.12	0.11	(0.10, 0.11)
25-74%	1061	2672	0.10	0.10	(0.09, 0.10)	7	97	0.06	0.05	(0.04, 0.07)	34	1297	0.09	0.08	(0.07, 0.09)	15	776	0.06	0.06	(0.05, 0.06)
$\geq$ 75%	3293	8229	0.32	0.31	(0.30, 0.31)	67	1045	0.57	0.58	(0.54, 0.62)	53	2175	0.14	0.14	(0.13, 0.14)	22	921	0.08	0.07	(0.06, 0.07)
location of a dosimeter																				
Inside	7114	16874	0.87	0.86	(0.86, 0.87)	75	1072	0.71	0.72	(0.68, 0.76)	87	2801	0.48	0.42	(0.40, 0.44)	47	1952	0.71	0.62	(0.59, 0.65)
Outside	568	1533	0.07	0.08	(0.07, 0.08)	7	97	0.07	0.07	(0.05, 0.08)	83	3236	0.46	0.48	(0.47, 0.50)	6	280	0.09	0.09	(0.07, 0.10)
Never	471	1156	0.06	0.06	(0.06, 0.06)	23	317	0.22	0.21	(0.18, 0.25)	12	655	0.07	0.10	(0.09, 0.11)	13	925	0.20	0.29	(0.27, 0.32)
Wearing a dosimeter																				
Always	9107	22120	0.82	0.83	(0.82, 0.83)	80	1084	0.60	0.60	(0.57, 0.64)	281	10296	0.64	0.64	(0.63, 0.66)	150	6841	0.55	0.50	(0.49, 0.51)
Sometimes	1676	3869	0.15	0.14	(0.14, 0.15)	41	542	0.31	0.30	(0.27, 0.34)	107	3802	0.25	0.24	(0.23, 0.25)	72	3804	0.26	0.28	(0.27, 0.29)
Never	343	803	0.03	0.03	(0.03, 0.03)	12	167	0.09	0.09	(0.07, 0.11)	48	1868	0.11	0.12	(0.11, 0.12)	50	3049	0.18	0.22	(0.21, 0.23)
Wearing a thyroid shield																				
Never	6679	17195	0.61	0.64	(0.63, 0.65)	62	826	0.47	0.46	(0.43, 0.49)	313	12042	0.72	0.75	(0.74, 0.76)	232	11790	0.85	0.86	(0.85, 0.87)
<25%	2004	4442	0.18	0.17	(0.16, 0.17)	15	211	0.11	0.12	(0.09, 0.14)	65	2123	0.15	0.13	(0.13, 0.14)	18	861	0.07	0.06	(0.06, 0.07)
25-74%	689	1508	0.06	0.06	(0.05, 0.06)	6	78	0.05	0.04	(0.03, 0.06)	17	556	0.04	0.03	(0.03, 0.04)	5	385	0.02	0.03	(0.02, 0.03)
$\geq$ 75%	1664	3641	0.15	0.14	(0.13, 0.14)	48	678	0.37	0.38	(0.35, 0.41)	38	1246	0.09	0.08	(0.07, 0.08)	17	658	0.06	0.05	(0.04, 0.05)
Wearing a lead goggle																				
Never	10060	24597	0.91	0.92	(0.91, 0.92)	111	1480	0.85	0.83	(0.80, 0.85)	400	14703	0.93	0.92	(0.92, 0.93)	266	13354	0.99	0.98	(0.97, 0.98)
<25%	494	1115	0.04	0.04	(0.04, 0.04)	10	148	0.08	0.08	(0.07, 0.10)	17	688	0.04	0.04	(0.04, 0.05)	1	126	0.00	0.01	(0.01, 0.01)
25-74%	170	370	0.02	0.01	(0.01, 0.02)	3	47	0.02	0.03	(0.01, 0.04)	4	184	0.01	0.01	(0.01, 0.01)	0	67	0.00	0.00	(0.00, 0.01)
$\geq$ 75%	309	704	0.03	0.03	(0.02, 0.03)	7	118	0.05	0.07	(0.05, 0.08)	10	390	0.02	0.02	(0.02, 0.03)	1	148	0.00	0.01	(0.01, 0.01)
Wearing lead gloves																				
Never	10280	24959	0.93	0.93	(0.93, 0.94)	115	1573	0.88	0.88	(0.86, 0.90)	401	14567	0.93	0.91	(0.91, 0.92)	264	13289	0.99	0.97	(0.97, 0.97)
<25%	452	1086	0.04	0.04	(0.04, 0.04)	11	150	0.08	0.08	(0.06, 0.10)	19	818	0.04	0.05	(0.05, 0.06)	2	140	0.01	0.01	(0.01, 0.01)
25-74%	97	244	0.01	0.01	(0.01, 0.01)	1	14	0.01	0.01	(0.00, 0.01)	6	299	0.01	0.02	(0.02, 0.02)	0	63	0.00	0.00	(0.00, 0.01)
$\geq$ 75%	202	498	0.02	0.02	(0.02, 0.02)	4	56	0.03	0.03	(0.02, 0.04)	6	283	0.01	0.02	(0.01, 0.02)	2	202	0.01	0.01	(0.01, 0.02)
Frequency of work separated from patients completely																				
100%	3686	10253	0.38	0.38	(0.37, 0.39)	47	716	0.41	0.40	(0.37, 0.43)	196	9350	0.55	0.59	(0.57, 0.60)	54	3095	0.22	0.23	(0.22, 0.24)
$\geq$ 75%	4540	12506	0.46	0.47	(0.46, 0.48)	42	674	0.37	0.38	(0.35, 0.40)	105	4300	0.29	0.27	(0.26, 0.28)	104	5674	0.42	0.41	(0.40, 0.43)
25-74%	1055	2772	0.11	0.10	(0.10, 0.11)	9	143	0.08	0.08	(0.06, 0.10)	29	1178	0.08	0.07	(0.07, 0.08)	57	3356	0.23	0.25	(0.23, 0.26)
<25%	496	1254	0.05	0.05	(0.04, 0.05)	16	261	0.14	0.15	(0.13, 0.17)	27	1138	0.08	0.07	(0.07, 0.08)	30	1569	0.12	0.11	(0.11, 0.12)
Frequency of work standing behind the shield wall																				
0%	426	991	0.06	0.06	(0.06, 0.06)	17	230	0.21	0.21	(0.18, 0.25)	24	740	0.11	0.11	(0.10, 0.12)	22	1160	0.11	0.11	(0.10, 0.12)
<25%	660	1542	0.10	0.09	(0.09, 0.10)	20	271	0.25	0.25	(0.21, 0.29)	30	951	0.14	0.14	(0.13, 0.16)	33	1806	0.16	0.17	(0.16, 0.18)
25-74%	1152	2696	0.17	0.16	(0.16, 0.17)	8	109	0.10	0.10	(0.08, 0.12)	34	1025	0.16	0.15	(0.14, 0.17)	55	3096	0.27	0.29	(0.28, 0.30)
$\geq$ 75%	4627	11305	0.67	0.68	(0.68, 0.69)	35	466	0.44	0.43	(0.39, 0.48)	128	3900	0.59	0.59	(0.57, 0.61)	97	4536	0.47	0.43	(0.42, 0.44)
Frequency of holding patients																				
0%	270	736	0.04	0.04	(0.04, 0.05)	17	213	0.21	0.20	(0.16, 0.23)	27	826	0.12	0.12	(0.11, 0.14)	10	336	0.05	0.03	(0.03, 0.04)
<10%	3406	8542	0.49	0.52	(0.51, 0.53)	38	513	0.47	0.48	(0.44, 0.52)	110	3271	0.49	0.49	(0.47, 0.52)	73	3389	0.34	0.32	(0.31, 0.33)
10-24%	1938	4417	0.28	0.27	(0.26, 0.28)	14	200	0.17	0.19	(0.15, 0.22)	40	1115	0.18	0.17	(0.15, 0.18)	54	2905	0.25	0.27	(0.26, 0.29)
$\geq$ 25%	1279	2838	0.19	0.17	(0.17, 0.18)	11	151	0.14	0.14	(0.11, 0.17)	49	1403	0.22	0.21	(0.20, 0.23)	75	3969	0.35	0.37	(0.36, 0.39)

S6 Table Result for radiological technologist, radiologist, dentist, and dental hygienist (1)

	Nurse					Doctor					Other					Assistant				
	N: obs	N: obs+imp	OBS mean	Pooled mean	95% CI	N: obs	N: obs+imp	OBS mean	Pooled mean	95% CI	N: obs	N: obs+imp	OBS mean	Pooled mean	95% CI	N: obs	N: obs+imp	OBS mean	Pooled mean	95% CI
Job_styr																				
	266	7726	2006.12	2007.34	(2006.09, 2008.58)	480	18961	2002.12	2001.68	(1999.69, 2003.68)	121	8925	2003.11	1997.22	(1996.61, 1997.83)	19	522	2000.26	2003.45	(2001.53, 2005.38)
Wearng a lead apron																				
0%	40	2236	0.17	0.29	(0.27, 0.30)	157	7018	0.36	0.37	(0.36, 0.38)	42	3940	0.39	0.44	(0.43, 0.46)	5	180	0.31	0.35	(0.29, 0.40)
<25%	19	673	0.08	0.09	(0.08, 0.10)	51	2166	0.12	0.11	(0.11, 0.12)	31	2567	0.29	0.29	(0.27, 0.30)	3	88	0.19	0.17	(0.12, 0.21)
25-74%	11	409	0.05	0.05	(0.05, 0.06)	27	1121	0.06	0.06	(0.05, 0.06)	8	640	0.07	0.07	(0.06, 0.08)	3	100	0.19	0.19	(0.14, 0.24)
$\geq$ 75%	169	4408	0.71	0.57	(0.55, 0.59)	204	8656	0.46	0.46	(0.45, 0.47)	27	1778	0.25	0.20	(0.19, 0.21)	5	154	0.31	0.29	(0.23, 0.36)
location of a dosimeter																				
Inside	183	4418	0.85	0.80	(0.79, 0.82)	196	7383	0.66	0.62	(0.61, 0.63)	62	3681	0.87	0.74	(0.72, 0.76)	10	274	0.83	0.80	(0.74, 0.86)
Outside	7	230	0.03	0.04	(0.03, 0.05)	63	2979	0.21	0.25	(0.24, 0.26)	2	686	0.03	0.14	(0.12, 0.15)	0	1	0.00	0.01	(-0.00, 0.01)
Never	25	842	0.12	0.15	(0.14, 0.17)	36	1581	0.12	0.13	(0.12, 0.14)	7	618	0.10	0.12	(0.11, 0.14)	2	66	0.17	0.19	(0.13, 0.25)
Wearing a dosimeter																				
Always	155	4274	0.59	0.55	(0.53, 0.57)	324	12754	0.67	0.67	(0.66, 0.68)	89	6869	0.75	0.77	(0.76, 0.78)	13	349	0.68	0.67	(0.61, 0.73)
Sometimes	79	2383	0.30	0.31	(0.29, 0.32)	119	4537	0.25	0.24	(0.23, 0.25)	19	1262	0.16	0.14	(0.13, 0.15)	4	103	0.21	0.20	(0.15, 0.25)
Never	28	1070	0.11	0.14	(0.13, 0.15)	42	1670	0.09	0.09	(0.08, 0.09)	11	794	0.09	0.09	(0.08, 0.10)	2	69	0.11	0.13	(0.09, 0.17)
Wearing a thyroid shield																				
Never	56	2386	0.21	0.31	(0.29, 0.32)	266	10798	0.56	0.57	(0.56, 0.58)	70	5902	0.59	0.66	(0.64, 0.68)	11	315	0.58	0.60	(0.55, 0.65)
<25%	24	674	0.09	0.09	(0.08, 0.10)	44	1624	0.09	0.09	(0.08, 0.09)	15	1026	0.13	0.11	(0.10, 0.13)	5	134	0.26	0.26	(0.20, 0.31)
25-74%	14	548	0.05	0.07	(0.06, 0.08)	20	759	0.04	0.04	(0.04, 0.04)	11	641	0.09	0.07	(0.06, 0.08)	0	1	0.00	0.00	(-0.00, 0.01)
$\geq$ 75%	169	4118	0.64	0.53	(0.52, 0.55)	144	5780	0.30	0.30	(0.29, 0.32)	23	1356	0.19	0.15	(0.14, 0.16)	3	72	0.16	0.14	(0.09, 0.19)
Wearing a lead goggle																				
Never	216	6331	0.82	0.82	(0.81, 0.83)	371	14039	0.78	0.74	(0.73, 0.75)	102	7348	0.86	0.82	(0.81, 0.84)	18	505	0.95	0.97	(0.94, 0.99)
<25%	20	529	0.08	0.07	(0.06, 0.08)	39	1697	0.08	0.09	(0.08, 0.10)	8	556	0.07	0.06	(0.05, 0.07)	1	17	0.05	0.03	(0.01, 0.06)
25-74%	8	224	0.03	0.03	(0.02, 0.03)	14	609	0.03	0.03	(0.03, 0.04)	6	508	0.05	0.06	(0.05, 0.07)	0	0	0.00	NaN	(NaN, NaN)
$\geq$ 75%	19	642	0.07	0.08	(0.07, 0.09)	50	2616	0.11	0.14	(0.13, 0.14)	3	513	0.03	0.06	(0.05, 0.06)	0	0	0.00	0.00	(-0.00, 0.01)
Wearing lead gloves																				
Never	260	7585	0.99	0.98	(0.98, 0.99)	412	16118	0.87	0.85	(0.84, 0.86)	106	7554	0.89	0.85	(0.83, 0.86)	18	501	0.95	0.96	(0.93, 0.99)
<25%	1	35	0.00	0.00	(0.00, 0.01)	32	1455	0.07	0.08	(0.07, 0.08)	7	559	0.06	0.06	(0.06, 0.07)	1	19	0.05	0.04	(0.01, 0.06)
25-74%	0	20	0.00	0.00	(0.00, 0.00)	12	572	0.03	0.03	(0.03, 0.03)	3	292	0.03	0.03	(0.03, 0.04)	0	1	0.00	0.00	(-0.00, 0.01)
$\geq$ 75%	2	85	0.01	0.01	(0.01, 0.01)	18	816	0.04	0.04	(0.04, 0.05)	3	520	0.03	0.06	(0.05, 0.07)	0	1	0.00	0.00	(-0.00, 0.01)
Frequency of work separated from patients completely																				
100%	26	1407	0.13	0.18	(0.17, 0.19)	236	11449	0.56	0.60	(0.59, 0.61)	55	5315	0.51	0.60	(0.58, 0.61)	5	159	0.36	0.30	(0.25, 0.36)
$\geq$ 75%	34	1445	0.17	0.19	(0.18, 0.20)	76	3162	0.18	0.17	(0.16, 0.17)	33	2549	0.31	0.29	(0.27, 0.30)	4	139	0.29	0.27	(0.21, 0.32)
25-74%	43	1704	0.21	0.22	(0.21, 0.23)	29	1143	0.07	0.06	(0.06, 0.07)	10	545	0.09	0.06	(0.05, 0.07)	4	180	0.29	0.35	(0.30, 0.39)
<25%	102	3170	0.50	0.41	(0.40, 0.42)	81	3207	0.19	0.17	(0.16, 0.18)	9	516	0.08	0.06	(0.05, 0.07)	1	44	0.07	0.08	(0.05, 0.12)
Frequency of work standing behind the shield wall																				
0%	63	1944	0.30	0.31	(0.29, 0.32)	45	1461	0.20	0.19	(0.18, 0.21)	8	377	0.13	0.10	(0.09, 0.12)	3	116	0.27	0.32	(0.25, 0.39)
<25%	55	1587	0.26	0.25	(0.24, 0.27)	50	1657	0.22	0.22	(0.21, 0.24)	6	284	0.10	0.08	(0.07, 0.09)	1	35	0.09	0.10	(0.05, 0.14)
25-74%	44	1381	0.21	0.22	(0.20, 0.23)	28	876	0.12	0.12	(0.11, 0.13)	15	865	0.25	0.24	(0.22, 0.26)	1	38	0.09	0.11	(0.06, 0.15)
$\geq$ 75%	48	1406	0.23	0.22	(0.21, 0.24)	104	3518	0.46	0.47	(0.45, 0.49)	32	2084	0.52	0.58	(0.56, 0.60)	6	174	0.55	0.48	(0.42, 0.54)
Frequency of holding patients																				
0%	23	760	0.11	0.12	(0.11, 0.13)	35	1281	0.15	0.17	(0.16, 0.18)	6	639	0.10	0.18	(0.15, 0.20)	2	94	0.18	0.26	(0.18, 0.34)
<10%	57	1795	0.28	0.28	(0.27, 0.30)	110	3693	0.48	0.49	(0.48, 0.51)	28	1540	0.45	0.43	(0.40, 0.45)	4	111	0.36	0.31	(0.23, 0.38)
10-24%	36	1202	0.17	0.19	(0.18, 0.20)	25	757	0.11	0.10	(0.09, 0.11)	17	998	0.27	0.28	(0.25, 0.30)	1	37	0.09	0.10	(0.06, 0.15)
$\geq$ 25%	90	2562	0.44	0.41	(0.39, 0.42)	59	1782	0.26	0.24	(0.22, 0.25)	11	434	0.18	0.12	(0.11, 0.13)	4	121	0.36	0.33	(0.26, 0.41)

S7 Table Result for nurse, doctor, other, and assistant (1)

	Radiological technologist					Radiologist					Dentist					Dental hygienist				
	N: obs	N: obs+imp	OBS mean	Pooled mean	95% CI	N: obs	N: obs+imp	OBS mean	Pooled mean	95% CI	N: obs	N: obs+imp	OBS mean	Pooled mean	95% CI	N: obs	N: obs+imp	OBS mean	Pooled mean	95% CI
Frequency of CT																				
Never	6699	16889	0.60	0.63	(0.62, 0.64)	112	1492	0.84	0.83	(0.80, 0.86)	278	13366	0.83	0.84	(0.83, 0.85)	44	10158	0.80	0.75	(0.74, 0.77)
<10	1230	2822	0.11	0.11	(0.10, 0.11)	9	129	0.07	0.07	(0.05, 0.09)	43	1880	0.13	0.12	(0.11, 0.13)	8	1703	0.15	0.13	(0.11, 0.14)
10-24	1168	2624	0.10	0.10	(0.09, 0.10)	5	64	0.04	0.04	(0.02, 0.05)	15	557	0.04	0.04	(0.03, 0.04)	3	1184	0.05	0.09	(0.07, 0.10)
25-49	706	1588	0.06	0.06	(0.06, 0.06)	3	41	0.02	0.02	(0.01, 0.03)	0	21	0.00	0.00	(0.00, 0.00)	0	190	0.00	0.01	(0.01, 0.02)
50-99	454	1044	0.04	0.04	(0.04, 0.04)	2	27	0.01	0.02	(0.01, 0.02)	0	21	0.00	0.00	(0.00, 0.00)	0	174	0.00	0.01	(0.01, 0.02)
≥ 100	882	1816	0.08	0.07	(0.06, 0.07)	3	40	0.02	0.02	(0.01, 0.03)	0	22	0.00	0.00	(0.00, 0.00)	0	65	0.00	0.00	(0.00, 0.01)
Frequency of C-Arm																				
Never	9128	22279	0.82	0.83	(0.83, 0.84)	127	1691	0.95	0.94	(0.93, 0.96)	332	15598	0.99	0.98	(0.98, 0.99)	55	12971	1.00	0.96	(0.95, 0.98)
<10	1393	3152	0.13	0.12	(0.11, 0.12)	7	91	0.05	0.05	(0.04, 0.07)	2	117	0.01	0.01	(0.00, 0.01)	0	27	0.00	0.00	(0.00, 0.00)
10-24	342	736	0.03	0.03	(0.03, 0.03)	0	2	0.00	0.00	(-0.00, 0.00)	1	46	0.00	0.00	(0.00, 0.00)	0	66	0.00	0.00	(0.00, 0.01)
25-49	120	266	0.01	0.01	(0.01, 0.01)	0	3	0.00	0.00	(-0.00, 0.01)	0	23	0.00	0.00	(0.00, 0.00)	0	86	0.00	0.01	(0.00, 0.01)
50-99	80	178	0.01	0.01	(0.01, 0.01)	0	4	0.00	0.00	(-0.00, 0.01)	1	56	0.00	0.00	(0.00, 0.01)	0	102	0.00	0.01	(0.00, 0.01)
≥ 100	75	170	0.01	0.01	(0.01, 0.01)	0	2	0.00	0.00	(-0.00, 0.00)	0	27	0.00	0.00	(0.00, 0.00)	0	220	0.00	0.02	(0.00, 0.03)
Frequency of fluoroscopy																				
Never	8135	19818	0.73	0.74	(0.73, 0.75)	70	907	0.52	0.51	(0.47, 0.54)	333	15609	0.99	0.98	(0.98, 0.99)	54	12977	0.98	0.96	(0.96, 0.97)
<1	1716	4040	0.15	0.15	(0.15, 0.16)	52	721	0.39	0.40	(0.37, 0.44)	2	119	0.01	0.01	(0.01, 0.01)	1	102	0.02	0.01	(0.01, 0.01)
1-4	629	1470	0.06	0.05	(0.05, 0.06)	10	136	0.07	0.08	(0.06, 0.09)	0	24	0.00	0.00	(0.00, 0.00)	0	51	0.00	0.00	(0.00, 0.01)
5-10	248	562	0.02	0.02	(0.02, 0.02)	1	16	0.01	0.01	(0.00, 0.01)	0	21	0.00	0.00	(0.00, 0.00)	0	86	0.00	0.01	(0.00, 0.01)
11-14	74	169	0.01	0.01	(0.01, 0.01)	0	0	0.00	0.00	(-0.00, 0.00)	1	64	0.00	0.00	(0.00, 0.01)	0	135	0.00	0.01	(0.01, 0.01)
≥ 15	336	722	0.03	0.03	(0.02, 0.03)	1	13	0.01	0.01	(0.00, 0.01)	0	27	0.00	0.00	(0.00, 0.00)	0	122	0.00	0.01	(0.01, 0.01)
Frequency of interventional therapy																				
Never	10437	25466	0.94	0.95	(0.95, 0.95)	105	1392	0.78	0.78	(0.75, 0.80)	335	15705	1.00	0.99	(0.99, 0.99)	55	13129	1.00	0.97	(0.97, 0.98)
<1	150	298	0.01	0.01	(0.01, 0.01)	13	182	0.10	0.10	(0.08, 0.12)	0	26	0.00	0.00	(0.00, 0.00)	0	55	0.00	0.00	(0.00, 0.01)
1-4	76	147	0.01	0.01	(0.00, 0.01)	4	58	0.03	0.03	(0.02, 0.04)	0	21	0.00	0.00	(0.00, 0.00)	0	65	0.00	0.00	(0.00, 0.01)
5-10	70	136	0.01	0.01	(0.00, 0.01)	9	121	0.07	0.07	(0.05, 0.09)	1	70	0.00	0.00	(0.00, 0.01)	0	74	0.00	0.01	(0.00, 0.01)
11-14	38	73	0.00	0.00	(0.00, 0.00)	2	26	0.01	0.01	(0.01, 0.02)	0	23	0.00	0.00	(0.00, 0.00)	0	116	0.00	0.01	(0.01, 0.01)
≥ 15	368	662	0.03	0.02	(0.02, 0.03)	1	14	0.01	0.01	(0.00, 0.01)	0	21	0.00	0.00	(0.00, 0.00)	0	35	0.00	0.00	(0.00, 0.00)
Duration of shift work																				
0	1958	9267	0.46	0.46	(0.46, 0.47)	38	1103	0.63	0.64	(0.61, 0.67)	387	14770	0.92	0.93	(0.92, 0.93)	222	12798	0.95	0.94	(0.93, 0.94)
<1	423	1963	0.10	0.10	(0.09, 0.10)	5	146	0.08	0.08	(0.07, 0.10)	11	390	0.03	0.02	(0.02, 0.03)	2	126	0.01	0.01	(0.01, 0.01)
1-2	567	2633	0.13	0.13	(0.13, 0.14)	4	113	0.07	0.07	(0.05, 0.08)	15	489	0.04	0.03	(0.03, 0.03)	3	184	0.01	0.01	(0.01, 0.02)
3-5	690	3140	0.16	0.16	(0.15, 0.16)	11	298	0.18	0.17	(0.15, 0.20)	4	154	0.01	0.01	(0.01, 0.01)	2	137	0.01	0.01	(0.01, 0.01)
6-9	354	1575	0.08	0.08	(0.07, 0.08)	2	57	0.03	0.03	(0.02, 0.04)	1	54	0.00	0.00	(0.00, 0.00)	5	380	0.02	0.03	(0.02, 0.03)
≥ 10	305	1367	0.07	0.07	(0.06, 0.07)	0	1	0.00	0.00	(-0.00, 0.00)	2	100	0.00	0.01	(0.00, 0.01)	0	30	0.00	0.00	(0.00, 0.00)

S8 Table Result for radilogical technologist, radiologist, dentist, and dental hygienist (2)

	Nurse					Doctor					Other					Assistant				
	N: obs	N: obs+imp	OBS mean	Pooled mean	95% CI	N: obs	N: obs+imp	OBS mean	Pooled mean	95% CI	N: obs	N: obs+imp	OBS mean	Pooled mean	95% CI	N: obs	N: obs+imp	OBS mean	Pooled mean	95% CI
Frequency of CT																				
Never	217	6301	0.82	0.82	(0.80, 0.83)	449	17880	0.94	0.94	(0.94, 0.95)	56	5676	0.54	0.64	(0.62, 0.66)	12	345	0.67	0.66	(0.61, 0.71)
<10	15	403	0.06	0.05	(0.05, 0.06)	12	417	0.02	0.02	(0.02, 0.03)	9	779	0.09	0.09	(0.08, 0.10)	1	27	0.06	0.05	(0.02, 0.08)
10-24	6	266	0.02	0.03	(0.03, 0.04)	10	315	0.02	0.02	(0.01, 0.02)	12	634	0.12	0.07	(0.06, 0.08)	3	91	0.17	0.17	(0.13, 0.22)
25-49	3	152	0.01	0.02	(0.01, 0.03)	0	19	0.00	0.00	(0.00, 0.00)	6	330	0.06	0.04	(0.03, 0.04)	1	30	0.06	0.06	(0.03, 0.09)
50-99	3	131	0.01	0.02	(0.01, 0.02)	3	120	0.01	0.01	(0.00, 0.01)	9	553	0.09	0.06	(0.05, 0.07)	1	28	0.06	0.05	(0.03, 0.08)
$\geq$ 100	22	472	0.08	0.06	(0.05, 0.07)	6	210	0.01	0.01	(0.01, 0.01)	12	935	0.12	0.11	(0.09, 0.12)	0	0	0.00	0.00	(-0.00, 0.01)
Frequency of C-Arm																				
Never	238	6826	0.89	0.88	(0.87, 0.90)	408	16556	0.85	0.87	(0.87, 0.88)	89	8132	0.86	0.91	(0.90, 0.93)	14	446	0.78	0.86	(0.81, 0.90)
<10	10	251	0.04	0.03	(0.03, 0.04)	33	1136	0.07	0.06	(0.05, 0.06)	6	236	0.06	0.03	(0.02, 0.03)	3	48	0.17	0.09	(0.05, 0.13)
10-24	8	234	0.03	0.03	(0.03, 0.04)	12	362	0.02	0.02	(0.02, 0.02)	4	181	0.04	0.02	(0.01, 0.03)	0	0	0.00	0.00	(-0.00, 0.01)
25-49	2	69	0.01	0.01	(0.01, 0.01)	8	255	0.02	0.01	(0.01, 0.02)	1	74	0.01	0.01	(0.00, 0.01)	0	0	0.00	0.00	(-0.00, 0.01)
50-99	2	72	0.01	0.01	(0.01, 0.01)	5	193	0.01	0.01	(0.01, 0.01)	1	88	0.01	0.01	(0.01, 0.01)	0	0	0.00	0.00	(-0.00, 0.01)
$\geq$ 100	6	273	0.02	0.04	(0.03, 0.04)	14	458	0.03	0.02	(0.02, 0.03)	3	198	0.03	0.02	(0.02, 0.03)	1	27	0.06	0.05	(0.02, 0.08)
Frequency of fluoroscopy																				
Never	168	5178	0.63	0.67	(0.66, 0.69)	378	14968	0.79	0.79	(0.78, 0.80)	85	7551	0.82	0.85	(0.84, 0.86)	15	444	0.83	0.85	(0.81, 0.90)
<1	21	476	0.08	0.06	(0.05, 0.07)	54	2088	0.11	0.11	(0.10, 0.12)	9	487	0.09	0.05	(0.05, 0.06)	2	47	0.11	0.09	(0.06, 0.13)
1-4	16	382	0.06	0.05	(0.04, 0.06)	22	843	0.05	0.04	(0.04, 0.05)	3	290	0.03	0.03	(0.03, 0.04)	0	0	0.00	0.00	(-0.00, 0.01)
5-10	19	498	0.07	0.06	(0.05, 0.08)	8	314	0.02	0.02	(0.01, 0.02)	2	146	0.02	0.02	(0.01, 0.02)	0	1	0.00	0.00	(-0.00, 0.01)
11-14	4	146	0.02	0.02	(0.01, 0.02)	5	218	0.01	0.01	(0.01, 0.01)	0	35	0.00	0.00	(0.00, 0.01)	0	1	0.00	0.00	(-0.00, 0.01)
$\geq$ 15	38	1045	0.14	0.14	(0.12, 0.15)	13	528	0.03	0.03	(0.02, 0.03)	5	399	0.05	0.04	(0.04, 0.05)	1	28	0.06	0.05	(0.02, 0.08)
Frequency of interventional therapy																				
Never	115	4532	0.43	0.59	(0.57, 0.60)	404	16236	0.84	0.86	(0.85, 0.86)	94	8249	0.90	0.93	(0.92, 0.93)	16	476	0.89	0.91	(0.88, 0.95)
<1	4	91	0.02	0.01	(0.01, 0.02)	22	805	0.05	0.04	(0.04, 0.05)	1	82	0.01	0.01	(0.01, 0.01)	0	1	0.00	0.00	(-0.00, 0.01)
1-4	8	205	0.03	0.03	(0.02, 0.03)	13	440	0.03	0.02	(0.02, 0.03)	2	144	0.02	0.02	(0.01, 0.02)	0	1	0.00	0.00	(-0.00, 0.01)
5-10	24	587	0.09	0.08	(0.07, 0.08)	12	452	0.02	0.02	(0.02, 0.03)	2	163	0.02	0.02	(0.01, 0.02)	0	1	0.00	0.00	(-0.00, 0.01)
11-14	5	173	0.02	0.02	(0.02, 0.03)	3	138	0.01	0.01	(0.01, 0.01)	0	47	0.00	0.01	(0.00, 0.01)	0	1	0.00	0.00	(-0.00, 0.01)
$\geq$ 15	110	2138	0.41	0.28	(0.26, 0.29)	26	888	0.05	0.05	(0.04, 0.05)	5	222	0.05	0.02	(0.02, 0.03)	2	42	0.11	0.08	(0.05, 0.11)
Duration of shirt work																				
0	17	2313	0.33	0.31	(0.29, 0.33)	186	11629	0.61	0.62	(0.61, 0.63)	37	7103	0.84	0.80	(0.79, 0.82)	6	378	0.60	0.74	(0.68, 0.79)
<1	0	24	0.00	0.00	(0.00, 0.01)	26	1532	0.09	0.08	(0.08, 0.09)	1	248	0.02	0.03	(0.02, 0.03)	0	0	0.00	0.00	(-0.00, 0.01)
1-2	5	794	0.10	0.11	(0.10, 0.12)	27	1543	0.09	0.08	(0.08, 0.09)	2	421	0.05	0.05	(0.04, 0.05)	0	0	0.00	0.00	(-0.00, 0.01)
3-5	9	1351	0.18	0.18	(0.17, 0.19)	46	2719	0.15	0.14	(0.14, 0.15)	3	720	0.07	0.08	(0.07, 0.09)	1	46	0.10	0.09	(0.06, 0.12)
6-9	13	1951	0.25	0.26	(0.24, 0.28)	7	468	0.02	0.02	(0.02, 0.03)	1	301	0.02	0.03	(0.03, 0.04)	1	36	0.10	0.07	(0.04, 0.10)
$\geq$ 10	7	1079	0.14	0.14	(0.13, 0.15)	12	897	0.04	0.05	(0.04, 0.05)	0	54	0.00	0.01	(0.00, 0.01)	2	52	0.20	0.10	(0.06, 0.14)

S9 Table Result for nurse, doctor, other, and assistant (2)
